# Situational vulnerability within mental healthcare – a qualitative analysis of ethical challenges during the COVID-19 pandemic

**DOI:** 10.1186/s12910-023-00910-3

**Published:** 2023-05-15

**Authors:** Mirjam Faissner, Anna Werning, Michael Winkelkötter, Holger Foullois, Michael Löhr, Jakov Gather

**Affiliations:** 1 Department of Psychiatry, Psychotherapy and Preventive Medicine, LWL University Hospital, Ruhr University Bochum, Alexandrinenstraße 1-3, Bochum, 44791 Germany; 2 Landschaftsverband Westfalen-Lippe (LWL), LWL-Dezernat für Krankenhäuser und Gesundheitswesen / LWL-PsychiatrieVerbund Westfalen, Münster, Germany; 3 Landschaftsverband Westfalen-Lippe (LWL), LWL-Klinikum Gütersloh, Gütersloh, Germany; 4 Fachhochschule der Diakonie, Bielefeld, Germany; 5 Institute for Medical Ethics and History of Medicine, Ruhr University Bochum, Bochum, Germany

**Keywords:** Discrimination, Mental healthcare, Psychiatry, Organizational ethics, Vulnerability

## Abstract

**Background:**

Mental healthcare users and patients were described as a particularly vulnerable group in the debate on the burdens of the COVID-19 pandemic. Just what this means and what normative conclusions can be derived from it depend to a large extent on the underlying concept of vulnerability. While a traditional understanding locates vulnerability in the characteristics of social groups, a situational and dynamic approach considers how social structures produce vulnerable social positions. The situation of users and patients in different psychosocial settings during the COVID-19 pandemic has not yet been comprehensively considered and ethically analyzed under the aspect of situational vulnerability.

**Methods:**

We present the results of a retrospective qualitative analysis of a survey of ethical challenges in different mental healthcare facilities of a large regional mental healthcare provider in Germany. We evaluate them ethically using a dynamic and situational understanding of vulnerability.

**Results:**

Difficulties in implementing infection prevention measures, restrictions of mental health services in favor of infection prevention, social isolation, negative health effects on mental healthcare users and patients, and challenges in implementing regulations on state and provider levels within the local specificities emerged across different mental healthcare settings as ethically salient topics.

**Conclusions:**

Applying a situational and dynamic understanding of vulnerability allows the identification of specific factors and conditions that have contributed to an increased context-dependent vulnerability for mental healthcare users and patients. These factors and conditions should be considered on the level of state and local regulations to reduce and address vulnerability.

**Supplementary Information:**

The online version contains supplementary material available at 10.1186/s12910-023-00910-3.

## Background

### Covid-19 and the situation of mental healthcare service users and patients in Germany

The impact of the COVID-19 pandemic on various social domains has been the subject of intense policy debates. While many statements and studies address different areas of life and problems, the situation of people who use mental healthcare services, who are in forensic or psychiatric facilities under involuntary commitment, or who live in mental healthcare facilities, residential facilities and care homes (hereafter: users and patients) has received much less attention [1–4].

Existing empirical studies indicate that people with mental illness were exposed to particular pressures during the pandemic. In terms of health outcomes, studies show an increased risk of infection and severe course, and an increased mortality compared to the general population [5–9]. This is particularly relevant considering the poorer health status of people with severe mental illness compared to the general population, which, according to the World Health Association, also stems from barriers to healthcare [10]. In addition, the pandemic placed a high burden on the mental well-being particularly of people with mental illness compared to the general population [11].

In terms of German mental healthcare, several changes have already been noted under pandemic conditions. Firstly, a reduction in treatment services, such as day clinics, group therapies, or self-help groups has been described [12]. Cancelled or postponed psychiatric appointments during the pandemic predicted higher depressive symptoms [12]. Furthermore, changes in practices of involuntary commitment for people with severe mental illness have occurred [1, 13, 14]. Difficulties in generally maintaining psychosocial services for mental healthcare users and patients while implementing infection controls were evident in various settings across the mental healthcare landscape. In line with this, the 2020 report from the National Agency for the Prevention of Torture notes specific challenges, such as ensuring social distancing measures and implementing quarantine measures during the COVID-19 pandemic in mental healthcare facilities [15]. Similar infection prevention challenges have also been reported in forensic psychiatric facilities [16]. Furthermore, while many restrictions have been loosened in a lot of societal domains, infection prevention measures continue to be in place in mental healthcare facilities.

People with mental illnesses have been identified as a “particularly vulnerable group” [17] in the ensuing debate. Accordingly, the statement of the German Ethics Council [18] on decision-making in the pandemic refers explicitly to the “special” vulnerability of different social groups, including people with mental illness. Just what this means and what normative conclusions can be derived from it depend largely on the underlying concept of vulnerability.

Even though vulnerability is a term much used in both traditional and contemporary bioethics, there is a lack of conceptual clarity [19–21]. It has mainly been discussed within research ethics, public health ethics [22], and, more recently, within clinical practice [23] and in aged care [24]. Traditional analyses aim at defining criteria to identify vulnerable individuals and then derive moral obligations. This mostly involves identifying *groups of people* who share features that put them under a heightened risk of an impaired capacity to consent, exploitation, and having one’s interest unjustly considered or harmed. Moral obligations may involve special protection through improvement of the informed consent procedures or exclusion from research [22].

However, critics of such *labeling approaches* have argued that identifying groups of people as vulnerable based on group-specific characteristics is stigmatizing, discriminatory, may deny agency to people identified as vulnerable, and may lead to paternalistic interventions [22, 25–28]. In addition, it has been suggested that the blanket exclusion of groups identified as vulnerable from clinical trials, for example, pregnant women or people under involuntary commitment, disadvantages the individuals affected because they do not benefit from research advances to the same extent as the general population [29, 30].

### Dynamic understandings of vulnerability

The German Ethics Council invokes an alternative concept of vulnerability in their report based on a situational and dynamic understanding. According to this, vulnerability is not inherent to any group defined by specific characteristics. Rather, vulnerable social positions arise depending on the context in specific “social, political, economic or also environmental constellations or interactions” [18]. In such an account, structural processes, such as discrimination, may contribute to the production of situational vulnerabilities.

The German Ethics Council’s use of the term is coherent with more recent theoretical works on vulnerability. Drawing on critiques of the traditional model [31], Luna [32] and further bioethicists [26] have developed a dynamic model of vulnerability for research ethics, public health, and the clinical context that uses the metaphor of “layers” [26]. Layers of vulnerability are dispositions that track an increased risk of suffering harm or disadvantage within a specific context. They may arise from different sources, both internal, such as age or a medical concern, and external, such as the social context, policies, or structural discrimination. The conditions that lead to the actualization of a vulnerability are called “triggers.” Different layers of vulnerability may coexist and be addressed independently of each other. “Layers of vulnerability with cascading effects” are those which may compound or generate further layers of vulnerability [26]. According to Luna, the concept of vulnerability is useful for bioethical evaluations, because normative obligations can be derived from sources and triggers of vulnerabilities [26].

To the best of our knowledge, no study has yet ethically evaluated the situational vulnerability of mental healthcare service users and patients during the COVID-19 pandemic.

### This study

In this article, we present the results of a retrospective qualitative analysis of a survey of ethical challenges during the COVID-19 pandemic among nursing and medical directors and facility managers of different mental healthcare facilities of the Regional Association of Westphalia Lippe (Landschaftsverband Westfalen-Lippe) in Germany. This analysis presents some of the different challenges faced in various mental healthcare settings during the first year of the pandemic. The study, thereby, considers that people with mental illness or mental health concerns use a diversity of services which range from outpatient departments, day-structuring services, and day clinics to inpatient psychiatric services, different types of residential facilities and care homes, and involuntary commitment in a psychiatric hospital or forensic psychiatric hospital.

This study aims, firstly, to provide an exploratory account of ethical challenges related to changes in mental healthcare facilities and practices during the COVID-19 pandemic. Secondly, it aims to ethically evaluate these challenges by using a dynamic and situational concept of vulnerability. More specifically, the goal of the study is to analyze contextual challenges in various mental healthcare settings in order to identify multiple layers of situational vulnerability that mental healthcare users and patients have experienced during the pandemic.

## Methods

We carried out a retrospective qualitative analysis of written survey documents. The survey documents were initially collected by the internal working group “Ethical dilemmas in psychiatry during the COVID-19 pandemic in institutions of the Regional Association of Westphalia Lippe.” This working group was established in the spring of 2021 to address ethical challenges in the context of the COVID-19 pandemic and develop practice-relevant recommendations as part of internal quality improvement measures.^1^ At the beginning of May 2021, it gathered ethical challenges in the everyday life of various mental healthcare facilities by means of a written survey of nursing and medical directors and facility managers in order to incorporate the experiences of on-site actors into the discussion. To this end, a prestructured written survey document was sent to the recipients via email. The survey documents were primarily used to inform the working group’s recommendations. They contained extended and rich descriptions of multiple ethical challenges from various mental healthcare settings, therefore, the authors decided to qualitatively analyze the data in-depth and use them for an empirically informed ethical analysis. The study presented here was approved by the Research Ethics Committee of the Medical Faculty of the Ruhr University Bochum (Reg. No.: 21-7290_BR). The authors follow the Standards for Reporting Qualitative Research proposed by O’Brien et al. [33].

### Data collection

The Regional Association of Westphalia Lippe is one of the largest mental healthcare providers in Germany, and comprises eleven hospitals for adult psychiatry, four hospitals for child and adolescent psychiatry, ten residential facilities and care homes for people with mental illnesses and disabilities, and six forensic psychiatric hospitals. The working group contacted all the nursing and medical directors of the hospitals, and all the facility managers of the residential facilities and care homes via emails at the beginning of May 2021. In this email, the recipients were invited to fill out a prestructured survey document on ethical challenges in their respective mental healthcare setting, after consulting with their staff. Participants were invited to fill in the survey document within four weeks. A reminder was sent after four weeks, with a second deadline after two weeks. The email informed the prospective participants about the survey’s aim to improve the quality of clinical and ethical decision-making during the pandemic by providing the different facilities with ethical recommendations. A prestructured survey document was attached to the email. The survey document was developed for this study by the working group based on reports from its members and the scientific literature. An English version translated by the authors can be found in the online supplement (Supp. 1). The survey contained a section to specify the participant’s current position and mental healthcare setting. The survey document was divided into six sections and inquired about different aspects in connection with the implementation of infection prevention measures: (1) effects on therapies according to guidelines, (2) experiences with contact restrictions, (3) coercive measures, (4) challenges in implementing infection control, (5) experiences with the vaccination program, and (6) experiences with the regulations on a state, provider, and facility level. The survey contained open and closed questions, and participants were invited to give written feedback to all sections using the free spaces available. The working group received a total of 27 out of 40 anonymized survey documents, which corresponds to a responder rate of 65%: 8 survey documents from adult psychiatry (2 medical directors, 5 nursing directors, 1 quality manager), 2 from child and adolescent psychiatry (1 medical director, 1 nursing director), 6 from forensic psychiatric hospitals (1 medical director, 5 nursing directors), 5 from residential facilities and 6 from care homes.

### Data analysis

A qualitative content analysis according to Kuckartz [34] was conducted. Data were analyzed using MAXQDA analysis software (MAXQDA 18 Standard Portable, VERBI Software GmbH, Berlin, Germany). The primary data analysis was conducted by two researchers conducting research into medical ethics and psychiatry: a cis-female *white* medical doctor with formal training in philosophy (MF), and a cis-female *white* medical student with a background in molecular biomedicine and a completed training as a peer support worker (AW). Both MF and AW have received training in qualitative methods by the Methods Centre of Ruhr University Bochum and conducted qualitative research prior to this project. They were supervised by JG (cis-male *white* consultant for psychiatry and psychotherapy with formal training in philosophy) who has supervised multiple qualitative research projects prior to this project. A structuring qualitative content analysis according to Kuckartz [34] was conducted, as it is a suitable approach for identifying and organizing themes that emerge from the data collected. The data analysis included seven steps: (1) MF and AW both read and commented on all survey documents individually and wrote summaries for each survey document; (2) they developed main categories under the supervision of JG after coding 20% of the documents, by combining inductive coding based on the content of the survey documents, with deductive coding based on the experiences of the working group members and the categories were discussed with JG; (3) all material was coded by MF and AW using the categories developed in step 3; (4) all text parts coded within the same category were reviewed together by MF and AW and the categories were assigned to superordinate themes together with JG; (5) the categories and superordinate themes were discussed with all members of the working group; (6) all survey documents were coded again by MF and AW; finally, (7) topics deemed ethically salient were chosen for discussion by all authors. Based on the ethical challenges identified and the discourse on vulnerable groups during the pandemic, the authors decided to ethically evaluate the former using a framework of dynamic vulnerability [26, 32]. The authors acknowledge that their social position, background, and experience with the mental healthcare system influences the interpretation of data. The research team was composed of individuals with diverse professional backgrounds (psychiatry, nursing sciences, health sciences, medical ethics, philosophy), and diverse relationships towards the mental healthcare system (personal experience, professional experience, research). Possible biases and implicit background assumptions were, therefore, discussed in the group and jointly reflected upon.

## Results

Five ethically relevant topics were identified. Selected verbatim quotations from the survey documents were translated from German into English by the first author and are inserted in italics.

### Topic 1: difficulties in implementing infection prevention measures in different mental healthcare facilities

Various difficulties in implementing infection prevention measures were reported. Firstly, social distancing and hygiene regulations could not always be implemented, as these were not invariably compatible with the facilities’ material structures. Several psychiatric hospitals and residential facilities, for example, were unable to provide isolation and quarantine areas with their own sanitary facilities. It was reported from one psychiatric hospital that employees shared the toilet with users and patients in quarantine due to the architectural structure.

In addition, challenges regarding room occupancy were reported in various clinical facilities. One forensic psychiatric hospital reported that seclusion rooms, intended for situations of acute danger to oneself and others, were used for admissions. This was necessary to bridge the time until a clear negative result of the COVID-19 test required upon admission occurred: “*The rooms are not originally furnished and have open sanitary facilities, with potential camera surveillance at the same time. Very limited furnishings with personal belongings and the uneasy feeling of being able to be observed from the outside at all times had to be endured by the patients housed there*” (forensic psychiatric hospital, nursing director no. 3). In some cases, due to overcrowding, it was not possible to offer patients a regular room after a clear COVID-19 test.

Finally, it was described that users’ and patients’ rights were restricted under infection prevention measures. One-on-one care was necessary in some residential facilities to maintain isolation. The constant accompaniment of people in their rooms was perceived by staff as a considerable intrusion into the users and patients’ privacy.

### Topic 2: restriction of mental health services in favor of infection prevention

Another theme was a restriction of psychiatric-therapeutic care in favor of infection prevention measures. Severely reduced therapy services were reported in various facilities during the first few months of the pandemic. Group therapy and cross-ward therapeutic offers were discontinued in (forensic) psychiatric hospitals, and were accompanied by an overall reduction in the therapy units available. The assisted living units reported the closure of day-structuring measures, which play an important role for residents in maintaining their day-night rhythm: “*The temporary (partial) closure of the day-structuring measures […] led to irritations and uncertainties: several users lost their regular day-night rhythm. After the facilities were opened again, some of them were no longer able to return to these services, which they had worked hard to acquire”*(residential facility, facility manager no. 3). Physiotherapy, occupational therapy, and specialist consultations were not available in some facilities for a longer period of time.

The infection prevention measures also led to various changes in the day-to-day care of users. The measures led to difficulties in “*optimally guaranteeing the provision of meals for the users during lockdown (snack bar closed, canteen closed, no cooking skills available)*” (residential facility, facility manager no. 1) in some residential facilities and care homes. In addition, several facilities eliminated communal meals; instead, all individuals ate in their rooms, despite the important function of communal meals in preventing loneliness and isolation.

Exposure training in the social home environment, which is an important part of preparing for discharge and assessing treatment outcomes in child and adolescent psychiatry, was suspended. Leaves were omitted in forensic psychiatric hospitals as part of correctional loosening or measures preparing for discharge. Finally, several facilities indicated that aftercare and outpatient follow-up treatments were severely limited due to a reduction in outpatient services.

### Topic 3: social isolation due to contact restrictions

All mental healthcare settings reported changes in visiting practices and restrictions on the premises. In this context, the regulations differed greatly between the facilities: whereas no blanket bans on visits had been imposed in one psychiatric hospital, in other hospitals, bans had been enforced with the use of security services. Strict regulations were implemented in one psychiatric hospital: “*The accesses to the site were closed off with construction fences. Information signs were posted. A general ban on visitors to the patients’ buildings was imposed. The great freedom of the park-like hospital grounds could not often be used properly*” (psychiatric hospital, quality manager). Drastic restrictions were also reported in a forensic psychiatric facility: “*significant restrictions on social contacts, some users and patients have not physically seen their relatives for more than a year*” (forensic psychiatric hospital, nursing director no. 1).

### Topic 4: negative health effects on users and patients

Negative effects of the different changes on the users and patients regarding therapeutic offers and contact restrictions were identified. The negative effects manifested in different ways in the various facilities. The main concern in child and adolescent psychiatry was social isolation that could lead to hospitalism.^2^ Children and adolescents suffered from not being able to visit their home environment and relatives. An increase of tensions was reported in acute psychiatric services as a consequence of users and patients’ inability to leave the ward to ensure contact restrictions. Nursing homes reported that isolation and a reduction of therapy services had led to a loss of psychosocial functioning for residents with dementia. Many of the latter had stopped recognizing their relatives during the pandemic. Social isolation was generally assessed as “*a high stressor for many residents*” (care home, facility manager no. 1). Physical (e.g. weight loss) and psychological effects (e.g. depressive moods) of residents with mental disabilities were described in the residential facilities as a consequence of changes in visiting and contact practices: “*The visiting ban manifested itself in increased withdrawal up to depressive moods, refusal to participate in in-house activities, refusal of individual one-to-one activities, and so on*” (residential facility, facility manager no. 3).

### Topic 5: competing regulations on different levels

Difficulties in dealing with the different and frequently changing regulations at a state level, regulations of the healthcare provider, and the specific local situation were reported. As a consequence, staff struggled to keep track of the current regulations and practices and reacted with an unwillingness and irritation to new adaptations: *“The changes, which were often communicated at very short notice, and the fact that regulations were often only valid for a short period of time, made it difficult to always pass on the correct guidelines. In some cases, these rapid changes led to a negative attitude among employees with regard to new regulations, and even to a refusal to accept and implement them”* (residential facility, facility manager, no. 3). Various parties also criticized the fact that the special situation and needs of people in mental healthcare facilities, especially in child and adolescent psychiatry, had not been taken into account at the state level: “*child and adolescent psychiatry has never been considered*” on the state level and only “*seldom on the provider level*” (child and adolescent psychiatry, medical director, no. 1). Somatic healthcare had generally served as a model for infection prevention measures. Special features of “*a psychiatric hospital with long hospitalization times, patients with social isolation and significant psychological stress due to the pandemic*” (psychiatric hospital, medical director no. 2) had not been considered. The fact that only a “*few concrete references [of the specifications] to daily life*” (psychiatric hospital, medical director no. 2) were made in the regulations was criticized. Reference was made to the potential for individual solutions and adaptations of the specifications within individual facilities, taking into account the experience of the employees, for example, in the implementation of digital communication with relatives.

## Discussion

The COVID-19 pandemic has created a challenging situation for society, policy makers, hospitals, and mental healthcare institutions. Regulations and practices had to be established within a short period of time to effectively protect the general population and groups designated as particularly vulnerable to COVID-19 infection. Against this background, various infection prevention measures were implemented in all mental healthcare facilities. Our results indicate that this has led to relevant changes in mental healthcare regarding the undesirable side effects of the ethically required protective measures. We will now discuss the results by identifying different layers of vulnerability.

### Layers of vulnerability in the context of infection prevention in mental healthcare facilities

Firstly, our analysis indicates that mental healthcare facilities faced difficulties in implementing the infection prevention measures recommended. The survey participants expressed their worry that spatial confinement and structural conditions contributed to an increased risk of coronavirus infection for people in mental healthcare facilities, especially at the time when no vaccination was available. This worry seems, to an extent, justified in the context of empirical studies showing an increased risk of infection for people in long-term care facilities [35], in combination with the poor physical health status of people with severe mental illness [10]. This indicates that an increased risk of coronavirus infection, associated with their housing situation and health status, was one layer of vulnerability for users and patients.

As a consequence, the facilities were confronted with the task of developing solutions for infection control in order to reduce the risks of infection under the circumstances given, such as overcrowding. In some cases, seclusion rooms were used for quarantine measures when admitting new patients to forensic psychiatric hospitals. These are intended for psychiatric emergency situations in which an acute danger to oneself or others can only be averted by such a measure. Because they do not constitute an appropriate therapeutic environment outside of these exceptional situations, such placement is problematic. The National Agency for the Prevention of Torture explicitly rejects the use of such rooms for quarantine or isolation measures [15].

In addition, mental healthcare facilities introduced contact restrictions to protect users and patients from infection and prevent outbreaks. These contact restrictions, while reducing infection risks, involved the social isolation of users and patients, with a simultaneous reduction in therapeutic or day-structuring services. The social isolation of users and patients in therapeutic settings is a particular burden because social relationships play a central role in the well-being of individuals. Our findings about the perceived psychological effects of social isolation, such as an increase in depressed mood and tensions, are consistent with findings in the international literature on the psychosocial effects of quarantine on individuals with mental illness [11].

Additionally, due to internal regulations, for example, regarding the use of space, daily routines and material equipment, users and patients only had very limited scope for action to deal with the negative effects of contact restrictions and a reduction of therapies. Thus, coping with the pandemic in a self-determined way was severely restricted – significantly more so than that of the general population, who were able to shape the concrete daily routines themselves within their homes. People with cognitive impairments, for example, were permanently accompanied within long-term care facilities to ensure contact restrictions. This may represent a restriction of the right to privacy, especially since, according to the Federal Working Group of Community Psychiatric Associations [36], the private character of bedrooms within long-term care facilities should be maintained as a “*retreat and protective space*.” Thus, the reduced scope to build individual coping strategies to deal with contact restrictions and the unavailability of therapeutic offers for service users and patients within prestructured institutional practices constituted another layer of vulnerability, which was triggered through the infection prevention measures, and involved harms such as the negative health effects of social isolation.

It is important to note here that mental healthcare facilities differ significantly from other institutions, such as somatic hospitals. Firstly, nursing facilities and residential homes are designed as long-term places of residence for users and patients and people with mental disabilities [36]. Similarly, individuals often stay significantly longer in psychiatric hospitals than in somatic hospitals.^3^ And individuals under involuntary commitment due to self-harm or danger to others are not free to choose their location, decide not to be hospitalized, or postpone psychiatric treatment [13, 14]. It is also unclear to what extent concrete alternatives were available for voluntary users and patients – especially since community-based services were also reduced or discontinued during the pandemic [36].

However, the regulations issued by the relevant federal and state authorities at the onset of the COVID-19 pandemic did not take into account the specificities of mental healthcare facilities. An analysis of these regulations conducted by Fasshauer and colleagues [38] indicates that the specific concerns of people with mental illness were not explicitly considered in the corresponding decrees: only three of the 16 German states issued separate regulations for mental healthcare facilities. According to Gomolla [39], indirect institutional discrimination occurs when ostensibly neutral legal regulations lead to group-specific disadvantages. It seems that the insufficient consideration of the special situation of mental healthcare institutions in federal and state regulations may have led to specific difficulties within these institutions and may, thus, have worked as a trigger for vulnerability.^4^

It follows that the justifiable effort to reduce the risk of coronavirus infection – to address one layer of vulnerability – resulted in other harms, such as increased psychological distress and negative health effects, in the context of contact restrictions within structures which inadvertently contribute to vulnerability by restricting the range of options for users and patients to cope with the new practices under corona regulations. We now discuss the ethical implications of our results.

### Implications

Our study indicates that users and patients of mental healthcare facilities were strongly affected by the impact of the COVID-19 pandemic. As the German Ethics Council (2022) writes: “Vulnerability is not merely a characteristic of affected individuals, but precisely the result of comprehensive constellations in which risk factors and protective factors interact in a complex manner. To a good extent, these constellations can also be influenced and produced.” Victor and colleagues argue that it is our obligation to “eradicate, minimize, and not exacerbate vulnerability” [26]. In our analysis, we have identified different layers and triggers of vulnerability of service users and patients during the pandemic. A dynamic model of vulnerability, in contrast to a more traditional understanding, allows one to see that these vulnerabilities are not *intrinsic* to mental illness or mental health conditions but produced by regulations that did not consider the specificities in mental healthcare settings.

What precisely does this entail for decision-makers on the level of local facilities, the healthcare provider, and the state? Luna [32] suggests ranking layers of vulnerabilities and to start by addressing first the most harmful, the most probable, and cascade layers if feasible, and to respect the preferences of the people concerned by fostering their autonomy [26]. Our analysis indicated that uniform regulations for somatic and mental healthcare settings at the state level led to a specific burden for mental healthcare users and patients. We, therefore, suggest that state regulations should be adapted for mental healthcare settings where necessary. Mental healthcare settings necessitate special attention to prevent indirect institutional discrimination. At the level of the healthcare provider, our results indicate that facilities benefitted from having enough room for adaptations to the local specificities. At the local level, our analysis has shown that organizational processes within facilities are ethically relevant. Clinical ethics committees may advise local decision-makers to find ethically justifiable and practically implementable regulations within set state regulations which are sensitive to the vulnerabilities identified [40]. Furthermore, service users and patients’ involvement can ascertain that their preferences and autonomy are respected in restructuring mental healthcare services and, as a recent review of the literature indicates, is associated with positive outcomes for service users [41].

### Limitations

Our study has some methodological limitations which need to be considered. Firstly, the working group conducted a written survey using a prestructured survey document. This means that the survey structure limited the range of possible answers. However, many participants took advantage of additional fields in the survey to add comments and explanations, and described their experiences beyond the topics mentioned in the survey. For this reason, we assume that we were able to capture many of the relevant ethical challenges.

In addition, only leading staff members (medical directors, nursing directors and facility managers) were invited to fill out the survey. Thus, the perspectives of users and patients and nonexecutives were not directly included. Since these groups are best able to provide information about their own experiences during the COVID-19 pandemic and have important knowledge gained from experience, their experiences and assessments should be identified in further studies.

Some limitations should also be considered regarding our application of the conceptual framework. Victor et al. [26] make suggestions and give examples of how to rank different layers of vulnerability. However, their account only gives a little normative guidance on deciding which layer is “most harmful,” especially in cases in which addressing one layer, such as high susceptibility to infection, means triggering other vulnerabilities, leading to harms such as the mental health burden of social isolation within institutions. As the framework stresses the importance of respecting the preferences of the people affected when addressing layers of vulnerability, we deem stakeholder participation to be important when ranking layers of vulnerability, which lies outside the scope of this article. Despite these limitations, our study provides important insights into some of the challenges faced by different mental healthcare settings during the first year of the COVID-19 pandemic and exemplifies how a dynamic understanding of vulnerability may be used for ethical analyses in empirical ethics research.

## Conclusion

Our qualitative analysis of a written survey on ethical challenges during the COVID-19 pandemic with executives within various settings of the mental healthcare system, including psychiatric hospitals, care homes, and residential facilities, shows the complexity of ethical decision-making during this time. The framework of dynamic vulnerability makes visible that the vulnerability of service users and patients is not *intrinsic* to their mental health conditions, but is *produced* by specific contextual, especially material and legal, conditions. This understanding helps to identify layers of vulnerability, address them specifically, and alleviate their impact. Such an understanding of vulnerability is helpful both for clinical and political decision-makers. Furthermore, in contrast to traditional labeling approaches, the framework of dynamic vulnerability stresses that the perspectives of those people concerned are crucial in ranking and addressing layers and triggers of vulnerability, and thereby highlights the importance of stakeholder participation.

Finally, our analysis suggests that mental healthcare institutions did not have the necessary material and human resources during the pandemic to adequately protect the people living in them or using their services from situational – *individual* – vulnerability. This is what the German Ethics Council has called “structural vulnerability,” i.e. an institution’s inability to maintain their functions to the degree required in a crisis [18]. Consequently, we stress the importance of providing mental healthcare facilities with the necessary personal and material resources to offer good care both in and after times of crisis.

## Electronic supplementary material

Below is the link to the electronic supplementary material.

Supplement 1: Survey

## Data Availability

We cannot share the research data publicly as this may compromise the privacy of research participants. The anonymized data can be made available by the corresponding author upon reasonable request.

## Footnotes

1 Members of the working group were (in alphabetical order): Ralf Borchert, Stephan Deimel, Mirjam Faissner, Lukas Finkam, Holger Foullois, Jakov Gather, Claus-Rüdiger Haas, Vera Herbst, Michael Löhr (head), Patricia Ohrmann, Mareike Schüler-Springorum, and Michael Winkelkötter.

2 Hospitalism refers to a combination of adverse mental and physical health effects children may suffer from in the context of prolonged institutionalization.

3 According to the German Association of Statutory Health Insurance Physicians [37], for example, the average length of stay in specialist departments for psychiatry and psychotherapy in 2017 was 73.3 days in preventive care and rehabilitation and 27 days in acute treatment, compared with an average of 21.8 and 6.4 days in internal medicine.

4 The structural discrimination that becomes visible here should be analyzed intersectionally, i.e. taking into account interlocking forms of marginalization, for example, based on socioeconomic position, race or sexual and gender diversity.
