# Supplementary material for: Situational vulnerability within mental healthcare – a qualitative analysis of ethical challenges during the COVID-19 pandemic
Source: BMC Med Ethics. 2023 May 15;24:31. doi: 10.1186/s12910-023-00910-3 (PMC10184624; doi:10.1186/s12910-023-00910-3)
Supplement: Supplementary file 1 — Supplement 1: Survey [file 12910_2023_910_MOESM1_ESM.docx]

**Online Supplement**

**Supplement 1: Survey**

(translated from German by the authors)

I am a member of the working group of

a) Nursing directors □

b) Medical directors □

c) Facility managers □

d) Other: _________________ □

in the field of

(a) adult psychiatry □

(c) child and adolescent psychiatry □

b) forensic psychiatry □

e) LWL care homes □

f) LWL residential facilities □

(g) Other: ____________________________ □

Please share your experiences and evaluations with us. You can use the free spaces to share specific cases that you remember as ethically challenging.

1. Do you remember any conflicts between the correct implementation of infection prevention measures and therapies according to guidelines?

(a) During the stay: [free space]

b) In preparation for discharge: [free space]

c) During follow-up care or outpatient treatment: [free space]

If so, please specify:

[free space]

2. What were your experiences with contact restrictions?

[free space]

a) What was, in your opinion, the impact on users (e.g. treatment, mental health) [free space]

b) What was, in your opinion, the impact on the institution? [free space]

3. Do you remember any coercive measures according to [German laws] with reference to corona? Please specify.

[free space]

4. Do you remember any difficulties in implementing infection control measures due to

a) structural factors / equipment? Please specify. [free space]

b) overcrowding? Please specify. [free space]

c) lack of staff? Please specify. [free space]

d) due to the behavior of patients/service users/residents? Please specify: [free space]

5. What were your experiences with the vaccination program?

[free space]

6. Did you encounter any challenges in dealing with corona regulations

a) on the state level? [free space]

b) on the provider level? [free space]

c) by the own facility? [free space]
